# Adolescent Admissions to Emergency Departments for Self-Injurious Thoughts and Behaviors

**DOI:** 10.1371/journal.pone.0170979

**Published:** 2017-01-26

**Authors:** Caterina Zanus, Sara Battistutta, Renata Aliverti, Marcella Montico, Silvana Cremaschi, Luca Ronfani, Lorenzo Monasta, Marco Carrozzi

**Affiliations:** 1 Institute for Maternal and Child Health – IRCCS “Burlo Garofolo”, Trieste, Italy; 2 Child and Adolescent Neuropsychiatry Service, ASS4 Medio Friuli, Udine, Italy; Central Institute of Mental Health, GERMANY

## Abstract

The objective of the present study was to describe the incidence and the characteristics of Self-Injurious Thoughts and Behaviors (SITBs), among adolescents aged 11–18 admitted, over a two year period, to all the Emergency Departments of a Region of North-eastern Italy through a comprehensive analysis of medical records. A two-step search was performed in the regional ED electronic database. First, we identified the cases that had been clearly diagnosed as SITBs by an Emergency Department physician. Secondly, suspect cases were detected through a keyword search of the database, and the medical records of these cases were hand screened to identify SITBs. The mean annual incidence rate of SITBs was 90 per 100,000 adolescents aged 11–18 years. Events were more frequent in females. Drug poisoning was the most frequently adopted method (54%). In 42% of cases a diagnosis of SITB was not explicitly reported by the physician. In 65% of cases adolescents were discharged within hours of admission. Only 9% of patients started a psychiatric assessment and treatment program during hospital stay. This research confirms the high incidence of SITBs among adolescents and highlights the difficulty in their proper diagnosis and management. Such difficulty is confirmed by the fact that only a few patients, even among those with a clear diagnosis, were sent for psychiatric assessment. Correct identification and management of SITB patients needs to be improved, since SITBs are an important public health problem in adolescence and one of the main risk factors for suicide.

## Introduction

Suicide is a major public health concern in adolescents, being the second leading cause of death in young people worldwide, [1] thus prevention initiatives aimed at young people, especially those at high risk, are strongly needed. [2]

The current most shared opinion is to consider suicide attempts, suicide ideations and self-injurious thoughts and behaviors (SITBs) all as elements of a spectrum of increased suicide risk [2] and, by convention, the majority of researchers and clinicians classify as “suicidal”, behaviors in which there is any evidence of any intent to die (i.e., at a “nonzero” level). [3]

Currently, SITBs are divided into suicidal and nonsuicidal: suicidal SITBs (e.g., suicide ideation, plans, attempts) are associated with any intent to die, whereas nonsuicidal SITBs (e.g., nonsuicidal self-injuries, suicide threats and gestures) are not. [3] Several studies show that nonsuicidal self-injuries (NSSIs), which by definition refer to “the direct and deliberate destruction of one’s own body tissue in the absence of lethal intent”, [3] are also significant risk factors for suicidal behavior. [4]

Among the known risk factors for suicidal behavior (mood, impulsivity, or disruptive behavior disorders, substance abuse, recent psychiatric hospitalization, family history of suicide, interpersonal violence, etc.), [5,6] previous suicide attempts and self-injurious thoughts and behaviors (SITBs) are consistently cited as among the strongest predictors of future suicidal behavior. [7]

Literature reports that the risk of suicide is significantly higher among adolescents engaging in self-injurious behaviors or referring suicide ideation. [8,9] Some studies estimate that in the year after an act of deliberate self-harm, the risk of suicide is 30 to 50 times higher than in the general population. [10] Even when enacted without suicidal intent, self-injury appears to be among the most robust predictors of suicidal thoughts and behaviors and some authors have proposed to consider it as an objective physical indicator of risk. [10]

In a recent meta-analysis on risk factors for potential suicidal behaviors (ideation, attempts, and death), prior SITBs are recognized as significant risk factors for suicide. [7] More specifically, prior suicide ideation is shown to be the strongest predictor of later ideation, while a history of NSSIs and suicide attempts confer the highest risk for later suicide attempts. A history of suicide attempts and suicide ideations is among the strongest predictors of suicide death. Also exposure to SITBs appears to be a strong predictor of suicide attempts, with effects comparable to factors such as prior suicidal ideation and past attempts.

Data from the literature show that, worldwide, the number of children and adolescents seen in emergency departments (EDs) and primary care settings for mental health problems has skyrocketed in recent years, with up to 23% of patients in both settings having diagnosable mental health conditions. [2,3,5,11–13] Moreover suicide attempts are one of the most common ED mental health presentations. [5]

Concerning the evaluation and management of children and adolescents with acute mental health problems (comprising SITBs and suicide ideation and/or attempts), three main issues are currently debated in literature: first, children and adolescents in psychiatric crisis are seen in general pediatric or medical EDs, which are crowded, noisy, high-stimulation environments, often with long waiting times and not enough private or quiet space; [6] second, most young people presenting with a psychiatric crisis are treated by pediatric emergency clinicians and staff who lack psychiatric training, or by adult psychiatric clinicians who lack training in the diagnosis and treatment of children and adolescents; [5,6,14,15] third, more than half of the youths presenting to the ED after a suicide attempt or another episode of deliberate self-harm do not receive a mental health assessment. [16,17]

Recently, there has been a call for action to improve the assessment and management of suicide risk in emergency medicine, [5,18–21] because EDs are considered by youths as a primary source of health care [22] and ED visits may represent a window of opportunity to identify youths at high risk for suicide, and to intervene effectively, preventing fatal and nonfatal suicidal behaviors. [23,24]

To date, in Italy, national epidemiological data on adolescent admissions to EDs for suicidal and nonsuicidal self-injurious thoughts and behaviors (which will be collectively referred to as SITBs for the remainder of the manuscript) are lacking and no national guideline exists for the management of adolescent suicide attempts in EDs.

The present study was conceived with the aim of contributing to the knowledge of this growing problem.

We retrospectively analyzed the medical records of adolescent admissions to all the EDs of our Region (Friuli Venezia Giulia, Italy), to identify both the cases with a clear diagnosis, and those that may have been misdiagnosed as unintentional accidents or not recorded in explicit terms. The main goal was to estimate the incidence of SITBs among adolescents admitted to the EDs of our Region, and to describe how such events are diagnosed and managed.

## Methods

All of the 21 EDs of Friuli Venezia Giulia (FVG) were involved in this retrospective study covering a two-year period (2005–2006). The study was coordinated by the Child Neurology and Psychiatry Department of the Institute for Maternal and Child Health—IRCCS “Burlo Garofolo” of Trieste. FVG is located in the North East of Italy and has a population of about 1,200,000 people. In this Region a standard electronic database is in use to report clinical information on all patients who access EDs. This database was first searched to identify adolescents aged between 11 and 18 admitted for SITBs to the EDs of the Region during the study period.

Since both literature and clinical practice suggest that self-injurious behaviors may be misdiagnosed as unintentional accidents or may not be recorded with specific terms such as “self-injurious”/“self-harming”/“suicidal behaviors” in the medical report, we designed a multi-step procedure in order to identify all possible cases.

A first pilot phase of the study was performed at the EDs of the two main cities of the Region (Udine and Trieste), as a training for the researchers and for the conceptualization and development of the search procedure: the admissions of adolescents to the EDs of the two cities were independently analyzed by two researchers (CZ, child neuropsychiatrist, and SB, psychologist) supervised by expert child neuropsychiatrists (SC and RA) and a two-steps procedure for the identification of SITBs was finally defined.

The first step (Step 1) was a keywords search. We used the following keywords: “suicide attempt”, “self-harming”, “self-injury”. As only some sections of the medical records were displayed in the database (*personal data*, with birth date, gender and admission date; *triage*, with cause of admission and severity code; *diagnosis*, with conclusive diagnosis and outcome), this allowed us to identify only the cases in which the keywords had been used in one of these sections of the medical records.

For Step 2 we used other, non-specific, keywords (S1 Table), chosen based on the terms used to describe self-injurious behaviors (such as poisoning/intoxication, cutting, precipitation…), and other causes of emergency admission known as possible misdiagnoses of self-injurious behaviors (trauma, accident). We also screened the accesses for altered mental states (psychomotor agitation, confusion) hypothesizing they could be clinical admission conditions for substance intake. The identified cases were analyzed more in detail by reading the entire medical record, and in particular the information reported in the clinical history and examination and the specialist’s evaluation, if performed. Using this procedure we found: a) cases in which the specific term “self-harm”/ “self-injurious”/ “suicidal”/ “suicide attempt” was reported in parts of the medical record which were different from those available in the electronic database; b) cases in which, even if not explicitly reported, the clinical information emerging from the history and examination was coherent with a diagnosis of SITBs, having satisfied the following inclusion criteria: a major criterion, such as the method of self-harm if highly specific to self-injurious behaviors (intentional precipitation, self-hanging, self-strangling), and one/or more than one minor criterion, including a less specific self-injurious method, like intoxication and cutting (e.g. use of psychotropic agents for intoxication, place and number of cuts for cutting), the potential dangerousness of the act (e.g. cocktail of high doses of psychotropic agents), the related circumstances (e.g. cocktail taken while alone), and the presence of trigger events reported as the cause of the behavior (family argument, school failure, emotional disappointment). As an example we report one of the clinical vignettes identified at the second step: “A 16-years-old girl arrived at the ED, accompanied by a friend, complaining of gastrointestinal symptoms and drowsiness which had appeared in the afternoon.” The diagnosis reported in the medical record was “poisoning”, no terms such as “self-harm”, “self-injury” or “suicide attempt” appeared in either the triage or the diagnosis sections of the electronic database; the case was identified at the second step using “poisoning” as a keyword: in the medical record it was reported that the girl had used a mix of psychotropic drugs, at a toxic dose, during a school break, and that she had done it because she felt sad for some recent stressful event.

All the cases identified in Step 2 as potential SITBs were assessed by the two researchers with a blinded evaluation. Disagreements regarding the labelling of a case were resolved by discussion between the two researchers and with the involvement of the two expert child neuropsychiatrists.

The complete medical records of all cases identified at either Step, were read and analyzed to collect more information on the events.

The incidence rate per 100,000 population was calculated as the mean of the two annual incidence rates, each calculated by dividing the number of acts/thoughts identified through our search for each of the two calendar years by the number of 11–18 years old adolescents living in the Region (74,138 in 2005 and 75,134 in 2006) derived from official population data (ISTAT, National Institute of Statistics). Incidence was calculated by age group (11–14 and 15–18 years) and by sex. Other informations collected were: 1) the methods used, 2) the time at which the SIBTs occurred: hour of the day and season, 4) the severity of the clinical conditions at admission and the duration of hospital stay, 5) the presence and the type of trigger events, 6) the presence of previous known psychiatric diagnoses, and 7) whether subjects were discharged from hospital after emergency mental health assessment.

The following variables collected from the ED records were organized in a database: age, sex, ED arrival time, severity of medical conditions at admittance (level of urgency of case based on triage color code: red = Life-threatening injuries requiring immediate medical attention, yellow = Non-life-threatening injuries requiring medical attention within 6 hours, green = Minor injuries, or white = Minor injuries not requiring medical care), methods of SITB, possible trigger events, presence of prior psychiatric diagnoses. The care and management of the cases were analyzed in terms of outcome (discharge, length of hospitalization), intervention by a mental health specialist and psychiatric assessment.

We conducted a descriptive analysis of the identified cases. We also studied possible differences between cases identified in Step 1 and Step 2. Multivariate logistic regression analysis was used to identify factors influencing the request for psychiatric assessment during the ED visit, considering the following independent variables: sex, hour, month, method, severity of medical conditions, known psychiatric diagnosis. A p-value <0.05 was considered as statistically significant. Statistical analyses were performed with Intercooled Stata 9.0 for Windows (StataCorp LP, 4905 Lakeway Drive, College Station, Texas USA, 2005). The study was approved by the technical and scientific committee (CTS) of the Institute for Maternal and Child Health—IRCCS “Burlo Garofolo” (RC 67/05).

## Results

**Fig 1:**
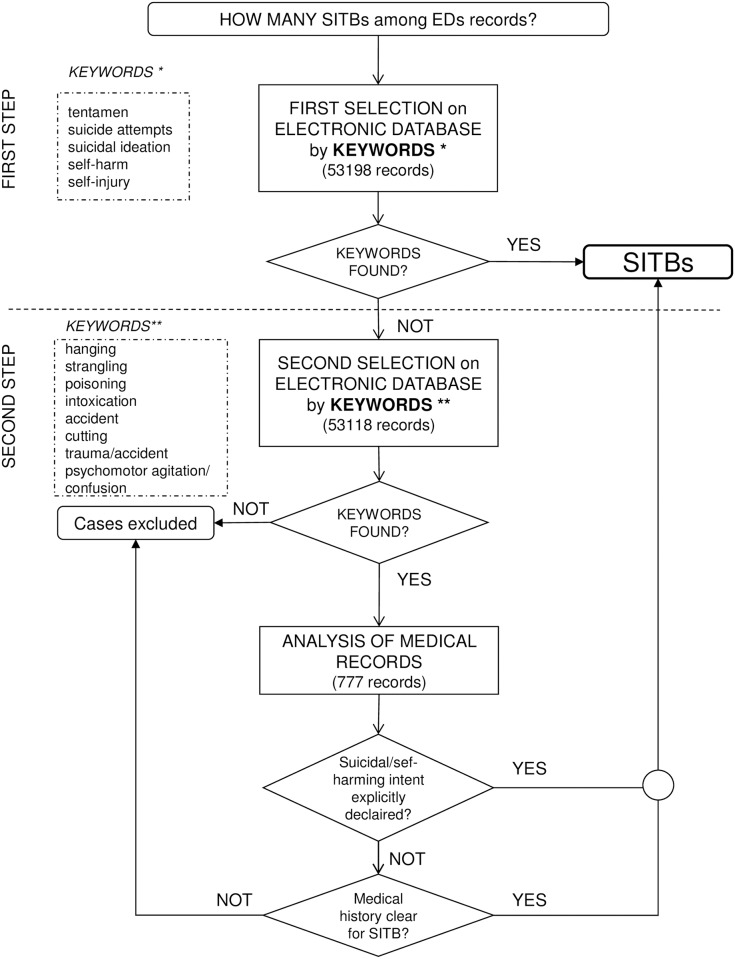
Flow-chart representing the two-steps procedure for the identification of SITBs among the cases visited in the EDs of the Friuli Venezia Giulia Region in the years 2005 and 2006.

The electronic records of 53,198 ED admissions of adolescents aged 11–18 years were analyzed (Fig 1). Seventy-eight episodes of SITBs were identified in Step 1. The Step 2 keyword search allowed us to identify further 777 accesses suspected to be SITBs, and the analysis of their medical records allowed us to detected 57 additional cases of SITBs. A total of 135 SITBs, over the two-year period of study, were thus finally identified; they involved 120 adolescents as there were some repeated acts. These cases accounted for 0.2% of the total 53,198 admissions to the EDs.

Overall, 58% of all the cases of SITBs were identified in Step 1: these were the cases in which the self-injurious intent was reported in the triage and/or in the final diagnosis sections of the medical report with a specific term such as “suicide attempt” / “self-harm”/ “self-injury”. The remaining cases (42%) were identified in Step 2, after analysis of the entire medical report. Among these cases, the most difficult to analyze were the episodes of cutting: when the terms “self-injury”/”self-harm” were not used, the self-injurious intent emerged only when the minor criteria were considered globally. These cases often required additional discussions between the researchers and the experts.

### Incidence rate and seasonality

The mean annual incidence rate of ED admissions for SITBs in adolescents aged 11–18 years in Friuli Venezia Giulia was 90 per 100,000. Incidence for adolescents aged 11–14 years was 27 per 100,000 (95% Confidence Interval: 16–41), and 155 per 100,000 (95% CI: 128–187) for those aged 15–18 years. The incidence rate was 64 per 100,000 (95% CI: 48–85) for males and 118 per 100,000 (95% CI: 94–145) for females, the latter being significantly higher (p = 0.001).

For both males and females, the highest number of cases was registered in the month of November (13/85 for females and 9/49 for males). If males and females are taken together, we notice a significantly higher incidence of events during autumn (September, October and November), with respect to the rest of the year (118/100,000 vs. 81/100,000; Fisher exact two-tailed test: p = 0.042) (Table 1). Comparing single seasons one to one, the only significant difference was found between autumn and summer (118/100,000 vs. 67/100,000; p = 0.022). When we stratified the data by sex, for females, despite autumn showing the highest incidence (159/100,000), no significant differences were found in comparisons between seasons. For males, winter and spring had the highest incidence, but a significant difference was only found between the lowest incidence during the summer and the rest of the year (32/100,000 vs. 75/100,000; p = 0.039).

**Table 1:** Seasonality of SITBs by sex among the cases visited in the EDs in the years 2005 and 2006: cases and, in parenthesis, incidence per 100,000.

Season	Females	Males	Total
Winter (December, January, February)	20 (109)	15 (79)	35 (94)
Spring (Mar, Apr, May)	18 (98)	13 (68)	31 (83)
Summer (June, July, August)	19 (104)	6 (32)	25 (67)
Autumn (September, October, November)	29 (159)	15 (79)	44 (118)
Total	86 (118)	49 (64)	135 (90)

### Characteristics of the episodes

Table 2 shows the main characteristics of the SITBs identified during the study period. The mean age of adolescents was 16 years and 10 months (SD = 1 yr and 7 m). The male/female ratio was 1.0:1.8.

**Table 2:** Characteristics of SITBs among the cases visited at the EDs in the years 2005 and 2006.

Variables analyzed	Step 1 (N = 78)	Step 2 (N = 57)	Step 1 and 2 (N = 135)
**Sex**			
• Male	30 (38%)	19 (33%)	49 (36%)
• Female	48 (62%)	38 (67%)	86 (64%)
**ED arrival time**			
• 1 a.m.– 8 a.m.	9 (12%)	5 (9%)	14 (10%)
• 9 a.m.– 4 p.m.	24 (31%)	17 (30%)	41 (31%)
• 5 p.m.– 12 p.m.	44 (57%)	35 (61%)	79 (59%)
**Modality used**			
• Cutting	20 (26%)	2 (4%)	22 (16%)
• Poisoning	32 (42%)	41 (72%)	73 (54%)
• Multiple	7 (9%)	1 (2%)	8 (6%)
• Other	18 (23%)	13 (23%)	31 (23%)
**Severity of conditions (triage code)**			
• White/green	32 (41%)	12 (22%)	44 (33%)
• Yellow	35 (45%)	37 (67%)	72 (54%)
• Red	11 (14%)	6 (11%)	17 (13%)
**Known psychiatric diagnosis**	**26 (33%)**	**23 (40%)**	**49 (36%)**
**Outcome of the visit**			
• Discharge	56 (72%)	33 (58%)	89 (66%)
• Hospitalization for < 36 hours	6 (8%)	11 (19%)	17 (13%)
• Hospitalization for > 36 hours	16 (20%)	13 (23%)	29 (21%)
**Request of mental health specialist assessment**	**33 (42%)**	**32 (56%)**	**65 (48%)**

The most frequently identified self-harm methods were poisoning (54% of cases: 63% in females and 39% in males) and cutting (16%, in both females and males) (Tables 2 and 3). Regarding poisoning, the substances most commonly used were drugs (79%) (Table 4), and in particular psychopharmaceutical drugs (Table 5).

**Table 3:** SITB cases visited in the EDs (n = 135): frequency and percentage of different methods used, including thoughts.

	Step 1	Step 2	Total	p-value*
Poisoning	32 (41%)	41 (72%)	73 (54%)	0.000
Cutting	20 (26%)	2 (4%)	22 (16%)	0.001
Contusion	8 (10%)	1 (2%)	9 (7%)	0.078
Suicide ideation / plan / threat / gesture / self-injury thoughts**	2 (3%)	6 (11%)	8 (6%)	0.070
Other	4 (5%)	3 (5%)	7 (5%)	1.000
Precipitation	4 (5%)	2 (4%)	6 (4%)	1.000
Poisoning + other	5 (6%)	1 (2%)	6 (4%)	0.401
Hanging	1 (1%)	1 (2%)	2 (1%)	1.000
Cutting + other	2 (3%)	0 (0%)	2 (1%)	0.505
Total	78 (100%)	57 (100%)	135 (100%)	

* p-values calculated comparing each category vs. all others, using a Fisher exact two-tailed test.

** According to Nock’s classification. [3]

**Table 4:** Poisoning among SITBs cases visited in the EDs: frequency and percentage (%) of cases for each substance used (n = 73).

Poisoning	Cases
Drugs	50 (68%)
Toxic substance	10 (14%)
Drugs + alcohol	5 (7%)
Alcohol	4 (5%)
Drugs + toxic substance	2 (3%)
Drugs + alcohol + toxic substance	1 (1%)
Alcohol + toxic substance	1 (1%)

**Table 5:** Drug poisoning among SITBs cases visited in the EDs: frequency and percentage (%) of cases for each drug used (n = 58).

Drugs	Cases
Psychopharmaceutical drugs	26 (45%)
“OTC” drugs*	11 (19%)
Other	4 (7%)
Antiepileptic drugs	3 (5%)
Psychoactive illicit substances	3 (5%)
Anti-migraine drugs	1 (2%)
Not specified	1 (2%)
Mixed Drugs**	9 (16%)

* Over-the-Counter drugs: medicines that may be sold directly to a consumer without prescription from a healthcare professional.

** Among the 9 cases who used “mixed drugs”, we found: 2 cases “psychopharmaceutical drugs + OTC”; 2 case “psychopharmaceutical drugs + Antiepileptic drugs”; 2 cases “OTC + Other”; 1 case “psychopharmaceutical drugs + Other”; 1 case “psychopharmaceutical drugs + OTC + Antiepileptic drugs”; 1 case “psychopharmaceutical drugs + OTC + Other”.

Terminology follows the World Health Organization criteria: http://www.who.int/substance_abuse/terminology/en/

Adolescents frequently engaged in SITBs late in the afternoon or in the evening: 59% occurred between 5 and 12 pm (Table 2).

Concerning the severity of the medical conditions at triage, 54% of cases were of medium severity (yellow code), meaning that the patient required medical attention within 6 hours because of potentially life-threatening injuries (Table 2), and 13% were severe (red code), requiring immediate medical attention.

Sixty-six percent of subjects were discharged soon after the visit, 13% was hospitalized for less than 36 hours, 21% for more than 36 hours or was transferred to another hospital.

Regarding possible trigger events, the information reported in the medical records was scarce: only in 27% of cases some recent event was mentioned, mainly related to family problems.

A prior psychiatric diagnosis was reported in 36% of cases, most frequently mood, personality or eating disorders. After ED access, a psychiatric assessment was requested in 48% of cases, and 9% of patients started a psychiatric assessment and management while staying at the hospital.

The multivariate logistic regression analysis carried out to identify factors influencing the request for psychiatric assessment during the ED visit, showed a statistically significant association only with known prior psychiatric diagnoses (Odds Ratio adjusted for variables listed in the Methods section: 8.7; 95% CI: 4.3–22.0; p<0.001).

The comparison between episodes detected in Steps 1 and 2 showed a statistically significant difference only for method (p<0.001): cutting was more frequent in cases identified in Step 1 (26% vs. 4% in Step 2) while poisoning was more frequent among cases identified in Step 2 (72% vs. 42% in Step 1).

## Discussion

To our knowledge, this is the first epidemiological study with full coverage of all emergency departments of an Italian Region, describing adolescent admissions to EDs for SITBs. In order to provide the most accurate evaluation of all SITBs, a two-step procedure was adopted for the identification of cases. Step 1 allowed us to collect clearly diagnosed SITB cases, while in Step 2 we focused on identifying misdiagnosed and undiagnosed SITBs.

According to our results, the mean annual incidence rate of SITBs in our Region is 90 per 100,000 adolescent aged 11–18 years, and such incidence rises to 155/100,000 if we only consider the 14 to 18 years of age group.

The 135 identified SITBs include eight cases of self-injurious thoughts. For these cases, the admission to the ED was the consequence of suicidal ideation/plan or threat/gesture expressed by the adolescent to parents or other familiar adults.[3] These cases were included on the grounds that they caused hospital admission, requiring clinical evaluation and entailing a clear diagnosis, and because self-injurious thoughts are in fact among the main risk factors for self-injurious behaviors and suicide (plan, attempts, death).

Regarding sex differences and methods of self-harm recorded in our study, the higher incidence rate found in females (64 per 100,000 males vs. 118 per 100,000 females) and self-poisoning as the most frequent method (54%), are in agreement with the literature. [25–28]

The variable regarding the method of self-harm was the only one for which a statistically significant difference was found between cases identified in Step 1 and in Step 2. Poisoning was the most frequent method of self-harm identified in both Step 1 and 2, but cuttings were mostly found in Step 1. However, while for poisoning, the self-injurious intent was quite easily identified by reading the medical records, the same was not true for cutting because medical records were much less detailed. Thus, as specified in the Results section, it is possible that we may have missed some of the cuttings with self-harming intent raising questions on whether poisoning is really the most frequent method used by self-harmers.

With respect to the distribution of cases over the 24-hours, our data show that adolescents acted mainly late in the afternoon or during the evening, a finding which is confirmed by the results of other authors. [1,29–30] We also found a higher frequency of SITBs in autumn and in particular in November and, for males, a lower frequency during the summer. Even if seasonality of suicide is a well-known phenomenon in literature, and has been described also for suicide attempts, findings appear to be quite mixed and contradictory. [31–34]

The analysis of the triage severity code revealed that in 50% of cases the clinical condition at admittance was of a potentially life-threatening injury, even if not severe enough to be classified as a “red code”. The fact that the clinical situation resolved easily and without long hospitalization (66% of youths was discharged soon after the visit) may have contributed to the under treatment of such cases. [22,27,35,36]

We searched for possible trigger events and psychopathologies. Probably as a result of the conciseness of ED medical records, such information was rarely available: only in 27% of cases we found mention of an event that could be interpreted as a possible stressor (mainly recent disputes within the nuclear family) and in 36% of cases a psychiatric diagnosis was reported as already known. Concerning the psychopathological aspects, different diagnoses were reported (mainly mood, anxiety, eating disorders, in order of frequency). These observations seem to be in agreement with most of the literature stating that self-harm in adolescence does not fit one stereotypical psychological description and different psychological subtypes are conceivable, [25] in which maladaptive coping strategies or emotional dysregulation can contribute to self-harming behavior. [2,37]

Regarding the mental health evaluation, only 9% of patients started psychiatric assessment and management while in the hospital and 66% of youths was discharged soon after the visit. Some authors report that background risk factors, such as social adversity and psychiatric history, have less influence on the assessment of suicide risk by an ED physician then the current mental state and the strong suicide intent. [10] According to our data, however, only the presence of a previous psychiatric diagnosis significantly increased the probability of requesting a mental health specialist evaluation. We hypothesize that a preexisting psychiatric diagnosis may induce the ED clinician to consider suicidality and self-harming behaviors, and to prescribe a consultation with a mental health specialist. We discussed these results with the ED physicians, and they confirmed their difficulty in exploring suicidality when faced with a self-injurious behavior, and in reporting such a diagnosis in the medical record.

This finding appears to confirm that, without an adequate and timely mental health assessment, the risk of suicidal behavior in several children and adolescents can easily go undetected [6,35,38].

According to the literature, presentation to hospital occurs in only about one in eight self-harming adolescents in the community [2,26] mainly because the physical consequences of these acts may not be severe enough to require medical attention. [39] Moreover, the difficulty to detect the self-injurious nature of such behaviors, which can be misdiagnosed as unintentional accidents, [39,40] and the heavy stigma surrounding suicide further affect reporting [11], thereby contributing to complicate the detection of these cases in retrospective studies. Misdiagnosis and difficulty in recognizing suicidal behavior are probably due to several reasons: first, the tendency of adults, both parents and clinicians, to deny and refuse the possibility of a self-inflicted injury or a suicidal intent in an adolescent; second, the fact that youths may not always disclose suicidal intent; third, the difficulties faced by physicians in the assessment and management of suicide risk in the busy setting of an emergency department. [14] In our study, such difficulties were especially obvious. In fact, 42% of the cases were identified only in the second step because the specific terms “self-injury”, “self-harm”, “suicide attempt” were not explicitly reported either in the triage or in the final diagnosis sections of the electronic medical records. There is growing literature on possible tools and practical guidelines for suicide risk assessment in EDs, [5,6,15,20,23,41] which could help improve the detection of SITB cases.

The comparison of epidemiological data from different studies is often problematic since the results reported in the literature can vary widely from country to country, and even within the same country. [1,27,42–44] Such variability seems to be mainly related to methodological issues that concern both the conceptual framework (definition of “suicidal” and “self-harming” behaviors) and the design (type of population analyzed, community or clinical).

Indeed, similar retrospective studies on adolescent admissions to EDs for suicide attempts can produce different results. Two large nationwide studies of US emergency department (ED) visits for attempted suicide and self-inflicted injury over a 16 and a 5-year period (with the longer study incorporating data from the shorter one) showed an incidence of 0.3 and 0.5 per 1,000 in the age group < 15 years and 3.1 and 3.3 for 1,000 in the age group 15–19, respectively. [45,46]

These incidence rates are higher than those documented by our research, but the results are only partially comparable since age ranges do not exactly overlap, and the data were obtained via chart review. It is interesting to note, however, that, even using different time scales, both papers find that the visits were highest among youths between the ages of 15 and 19; the authors conclude that this finding is consistent with evidence that suicide is the third leading cause of death among 15–24 year-olds.

A similar incidence (3.0 for 1,000) in terms of self-harm diagnostic codes, was documented by another, more recent, 9-year retrospective cohort study of adolescents aged 15–17 years from Alberta (Canada). [38]

Few national data on self-injurious behaviors are currently available for Italy, especially in ED setting. Researches rely either on epidemiological surveillance at school, [47,48] or on cases of death for suicide. [49–51] The only available ED based data for Italy refer to the city of Padua, as part of a WHO/EURO multicenter study, and show a prevalence rate per year of 17 per 100,000 for males and 188 per 100,000 for females (aged 15–19 years), [52] and to the city of Trento in which the ongoing “suicide prevention program” has identified 55 cases of suicide attempts (20 males and 33 females) in the 15–19 age group (between 2009 and 2014) (incidence of 3.3 per 100,000). [53]

It was not possible to classify the identified cases into levels of severity, as we could not link them to information on long-term outcomes. The clinical conditions at admission (triage severity code) only describe the severity of the medical consequences of the act, but do not necessarily reflect the severity of the risk of suicide. Possible indicators of severity could be the repetition of the act (12 out of the 120 subjects, 10%, in the following year) and the result of the psychiatric evaluation, but this was performed only in a minority of cases. In line with data in literature, our results confirm lack of mental health assessment to be a critical aspect of SITBs prevention, especially when patients do not have a previous psychiatric diagnosis.

Regarding incidence rates, which are lower in our series compared to literature, we can make some assumptions: the methods used for the classification and identification of the cases are different, as are the size of the analyzed geographical area and the healthcare setting, plus there is wide variability in social and demographic variables. Italy has a “universalistic” healthcare system with equal access to health services for all citizens and community-based mental health centers scattered across the Region that may have intercepted part of the potential users.

Nevertheless, the low number of mental health assessments performed in EDs on subjects with STIBs remains a critical issue.

We believe our study has several strengths: i) the involvement of all the 21 EDs of the Region using a regional standard electronic database, ii) a two-step procedure that included, in step 2, the screening of all suspect medical records, and iii) its contribution, as the most comprehensive data set analyzing SITBs admission to EDs, to the evaluation of the incidence and the characteristics of SITBs in Italy.

This study however, also has several limitations that need to be mentioned. The main one is certainly the retrospective nature of the analysis of ED medical records, in which information may be scarce and incomplete. In particular, as previously mentioned, our data do not allow for a systematic appraisal of suicide intentionality nor for the identification of the trigger events and/or reasons for the acts. Likewise, it was not possible to classify the identified cases in terms of severity, even if only cases in which the nature of the SITB was clear, although not always explicitly reported, were included in the analysis.

We also need to mention that the most difficult category to evaluate, in terms of methods, was cutting, due to the lack of detailed information on the history of the episodes reported in the medical records. We are aware that, in particular with regards to episodes of cutting, we may have missed cases which were not explicitly reported as SITBs and could have, therefore, underestimated the incidence rates.

Finally, the data collection for the years 2005 and 2006, was conducted in 2007 in the context of a project supported by the Italian Ministry of Health. At the end of the project, the results were delivered to the MoH and discussed with the staff of the EDs. Only recently, thanks to new funding for research on self-harming and suicidal behaviors in adolescents in schools, were we able to complete the analysis and produce the present manuscript. However, we have no reason to doubt that these results are still relevant, considering that little has been done in recent years to improve the capacity of ED staff to detect, report and refer adolescents accessing the emergency department for SITBs.

## Conclusions

Our data highlight the importance of improving the ability of ED staff to identify and report SITBs, to help evaluate subsequent readmissions, considering that the repetition of the act is an important risk factor. Since the ED is often the first and only health care contact point, adolescents accessing the service for SITBs need to be recognized, diagnosed, and given appropriate treatment to help prevent them from attempting suicide in the future.

Even if SITBs are a well-known important public health problem and self-harming and suicide attempts are reported in literature as the main risk factors for suicide, no national guideline is currently available for the management of suicide attempts in EDs, and no specific intervention has been implemented in the past ten years in our Region to improve the clinical practice in this field.

Future directions should focus on appropriate training programs for the early identification in EDs of patients at high risk for suicide [24]. A growing number of studies focus on tools and practical guidelines for suicide risk assessment in EDs to facilitate triage of young people who harm themselves. [5,16,54,55].

We believe the ED staff should receive training in the provision of basic mental health care, [27] and that stronger collaboration is required between ED physicians and mental health specialists [24] to ensure effective care transition to outpatient settings and to reduce the chances of a relapse. [56]

## Supporting Information

S1 Table Keywords and criteria used in Step 2 to identify self-injurious thoughts and behaviors among accesses to the Emergency Departments of Friuli Venezia Giulia (Italy), not clearly identified in Step 1. (PDF)
