# Supplementary material for: Adolescent Admissions to Emergency Departments for Self-Injurious Thoughts and Behaviors
Source: PLoS One. 2017 Jan 26;12(1):e0170979. doi: 10.1371/journal.pone.0170979 (PMC5268645; doi:10.1371/journal.pone.0170979)
Supplement: S1 Table — (PDF) [file pone.0170979.s001.pdf]

S1 Table. Keywords and criteria used in Step 2 to identify self-injurious thoughts and behaviors among accesses to the Emergency Departments of Friuli Venezia Giulia (Italy), not clearly identified in Step 1.

► suicide attempt / suicidal ideation / self-harm / self-injury: all considered as SITB
► hanging / strangling: all considered as SITB
► poisoning / intoxication<br>combined evaluation of the following criteria: potential dangerousness of the act, way of arrival (emergency service, car, alone), day and hour of the event (eg. working day, afternoon hours, etc.), circumstances of the episode (eg. acted out by itself), trigger events reported as the cause of the act (eg. family argument, scholastic failure, emotional delusion).<br>• Examples of cases included: drinking of alcohol at home, being alone, after a trigger event, with a cocktail of substances, repeated alcohol intoxications;<br>• Examples of cases excluded: accidental episodes, drinking with friends at a party.
► accident / trauma<br>combined evaluation of the following criteria: modalities and circumstances of the episode; repetitiveness; non-use of the safety devices.<br>• Examples of cases included: modality and circumstances of the act (being drunk, being alone, after a trigger event, repeated several times in the same period, non-use of the safety devices;<br>• Examples of cases excluded: non-repeated episode, absence of suspicious circumstances.
► cutting<br>combined evaluation of the following criteria: number and place of the cuts, circumstances of the episode.<br>• Examples of cases included: multiple cuts, in the arm, forearm, wrist, hand, leg, neck, foot<br>• Examples of cases excluded: unusual site for a self-injurious act (eg. forehead).
► confusion / psychomotor agitation<br>declaration of a suicidal / self-injurious thought (ideation/planning/threatening), or any self-injurious behavior.
